# Use of Stroop Test for Sports Psychology Study: Cross-Over Design Research

**DOI:** 10.3389/fpsyg.2020.614038

**Published:** 2020-12-07

**Authors:** Shinji Takahashi, Philip M. Grove

**Affiliations:** ^1^Faculty of Liberal Arts, Tohoku Gakuin University, Sendai, Japan; ^2^School of Psychology, The University of Queensland, St Lucia, QLD, Australia

**Keywords:** inhibitory function, random effect, individuality, experimental design, statistical power

## Abstract

**Background:** In sports psychology research, the Stroop test and its derivations are commonly used to investigate the benefits of exercise on cognitive function. The measures of the Stroop test and the computed interference often have different interclass correlation coefficients (ICC). However, the ICC is never reported in cross-over designs involving multiple variances associated with individual differences.

**Objective:** We investigated the ICC of the Stroop neutral and incongruent tests and interference (neutral test—incongruent test), and reverse Stroop task using the linear mixed model.

**Methods:** Forty-eight young adults participated in a cross-over design experiment composed of 2 factors: exercise mode (walking, resistance exercise, badminton, and seated rest as control) and time (pre- and post-tests). Before and after each intervention, participants completed the Stroop neutral and incongruent, and the reverse-Stroop neutral and incongruent tests. We analyzed for each test performance and interference and calculated ICC using the linear mixed model.

**Results:** The linear mixed model found a significant interaction of exercise mode and time for both the Stroop and reverse-Stroop tasks, suggesting that exercise mode influences the effect of acute exercise on inhibitory function. On the other hand, there was no significant effect of exercise mode for both the Stroop and reverse-Stroop interference. The results also revealed that calculating both the Stroop and reverse-Stroop interference resulted in smaller ICCs than the ICCs of the neutral and incongruent tests for both the Stroop and reverse-Stroop tasks.

**Conclusion:** The Stroop and reverse-Stroop interferences are known as valid measures of the inhibitory function for cross-sectional research design. However, to understand the benefits of acute exercise on inhibitory function comprehensively by cross-over design, comparing the incongruent test with the neutral test also seems superior because these tests have high reliability and statistical power.

## Introduction

Several studies have demonstrated that exercise has beneficial effects on brain structure and cognitive function (Colcombe et al., 2006; Pedersen et al., 2009). For example, regular exercise can increase brain volume of older people (Colcombe et al., 2006). To elucidate the mechanism of how exercise affects the structure and function of the brain, researchers have investigated intensity, duration, and mode of exercise (Lambourne and Tomporowski, 2010; Voss et al., 2011; Chang et al., 2012). The Stroop task (Stroop, 1935) which can measure the inhibitory function is extensively applied in research (Etnier and Chang, 2009). The Stroop task is commonly composed of a neutral test, a congruent test, and an incongruent test. For the neutral and congruent test, individuals are required to name the color of irrelevant letters (e.g., XXXX), a color patch, or the corresponding color word (e.g., “Red” is printed in red ink). In the incongruent test, individuals suppress reading the meaning of the word and respond to the color of the ink which is not matched to the color name (e.g., “Red” is printed in blue ink). Typically, the incongruent test yields a longer response time relative to both the neutral and congruent test. The delay of the response in the incongruent test is called “Stroop effect,” and it is associated with activation in brain regions (e.g., prefrontal cortex, anterior cingulate cortex) associated with the control executive function (Ruff et al., 2001; Zysset et al., 2001; Song and Hakoda, 2015).

The reverse-Stroop task is a derivation of the Stroop task employed to measure inhibitory function. During the reverse-Stroop task, individuals are asked to respond to the word while ignoring the color of the text rather than identifying the color and ignoring the word. Although the reverse-Stroop task is thought to measure inhibitory function as well as the Stroop task, there are the results which the brain regions associated with the reverse-Stroop task differs from those of the Stroop task (Ruff et al., 2001; Song and Hakoda, 2015). The reverse-Stroop task has been used by researchers to investigate how acute exercise influences executive function (Tsukamoto et al., 2016a,b). These studies have obtained large effect sizes with relatively small samples, suggesting that the reverse-Stroop task is sensitive to the effect of exercise.

Although the Stroop and reverse-Stroop tasks are adopted to assess the inhibitory function, there is still debate around the method of measurement (Scarpina and Tagini, 2017). Scarpina and Tagini (2017) systematically reviewed studies in which used the Stroop task, suggesting that researchers should report not only test performance (e.g., reaction time or the number of correct responses) but also the Stroop interference which is defined as the difference between the neutral/congruent test and the incongruent test. The neutral and congruent tests which do not involve cognitive conflict are categorized as a test of the information processing (Chang et al., 2012). Given that the incongruent test might be affected by information processing constraints, it seems that the interference which partials out the contribution of information processing is a better index than the incongruent test. Indeed, several studies reported that the Stroop interference is associated with specific structures of the brain, cortical activation, and psychological arousal (Takeuchi et al., 2012; Byun et al., 2014; Song and Hakoda, 2015), suggesting that the interference is a valid and useful measurement of the inhibitory function.

On the other hand, there is a possibility that incongruent test performance is a better measure of inhibitory function than interference in complex experimental research designs. This is because the intraclass correlation coefficient (ICC) associated with incongruent performance could be higher than for interference (Siegrist, 1997; Strauss et al., 2005; Hedge et al., 2018). Specifically, in cross-over or mixed designs (Barnhart et al., 2007; Nakagawa and Schielzeth, 2010), higher ICC enhances statistical power. Although a number of previous studies investigated the reliability of the Stroop task using ICC (Franzen et al., 1987; Kozora et al., 2004; Strauss et al., 2005; Wallman et al., 2005; Portaccio et al., 2010; Mohammadirad et al., 2012; Register-Mihalik et al., 2012; Bajaj et al., 2015; Martínez-Loredo et al., 2017), the manners of the Stroop task and the assessment of interference were varied and how to test ICC has been not formatted yet (Parsons et al., 2019). Therefore, the ICC about the Stroop task and its interference seems to has not been adequately examined.

Previous studies involving test-retest designs revealed that each test of the Stroop task showed a higher ICC, than Stroop interference (Siegrist, 1997; Strauss et al., 2005; Hedge et al., 2018). ICC is defined as the ratio of the variance between participants and the sum of the between participants and the residual variances (Shrout and Fleiss, 1979). Hedge et al. (2018) also explained that calculating interference did not affect the residual variance but it reduced the variance associated with individual differences. In experimental research, the effect of exercise on the inhibitory function may be masked due to low ICC and statistical power. Therefore, the Stroop incongruent test performance might be better suited to experimental research than the Stroop interference.

If calculating the interference selectively reduces the variance between participants, the ICC of the Stroop interference might decrease more substantially in a cross-over design. Test-retest research measures of the Stroop task involve only two observations per participant. On the other hand, cross-over designs involve at least four measures per participant (e.g., experimental condition and control condition × pre-test and post-test). Given that the positive impact of exercise on the inhibitory function is small to medium (Lambourne and Tomporowski, 2010; Voss et al., 2011), cross-over designs need to enhance statistical power using measurements with high ICC. However, to the best of our knowledge, no previous investigations have reported ICC of the Stroop test performance and interference for cross-over designs. Therefore, we investigated the ICC of the Stroop task in a cross-over design investigating the effect of exercise on inhibitory function.

One of the reasons why ICC in cross-over design research has not been reported is concerned with statistical analysis. The ICC is commonly calculated using the outputs of one- or two-way analysis of variance (ANOVA) in which one factor is participants. The ANOVA uses the moment method to estimate variance components. This method cannot directly distinguish the variance between participants and the residual variance. Even in a simple test-retest design with both between participants variance and the residual variance as random effects, the moment method cannot distinguish between the two variances. However, the moment method estimates the between participants variance by subtracting from the total random effects’ variance (the sum of the variance between participants and the residual) to the residual variance (Shrout and Fleiss, 1979). Therefore, this method can yield a negative ICC when a sum of variance components of individual differences is smaller than a residual variance, which is substantially meaningless. This disadvantage is a challenge to apply ANOVA in cross-over designs in which there are multiple variances associated with individual differences.

To be able to calculate ICC in a cross-over design, Nakagawa and Schielzeth (2010) and Hedge et al. (2018) suggest using the linear mixed model (LMM), also known as a multilevel model or a hierarchical linear model. The LMM, unlike ANOVA, can estimate each parameter using maximum likelihood (ML) or restricted maximum likelihood (REML), computing multiple variances associated with individual differences separately from the residual variance. Brouwer et al. (2012) and Demetrashvili et al. (2016) demonstrated that the ICC can be calculated using the LMM even in complicated research designs which have multiple variances associated with individual differences. We aimed to calculate the ICC for the Stroop task in a cross-over design investigating an acute exercise effect on inhibitory function and to consider the ICCs’ influence on revealing the effect of acute exercises. We also calculated ICC of the reverse-Stroop task. As described above, although the reverse-Stroop task is a useful measurement, no previous reports have reported the ICC for reverse-Stroop tasks.

We expected that individual tests will show higher ICCs than the interferences for both of the Stroop and reverse-Stroop tasks, and each test with higher ICCs may be more likely to reveal the effects of exercises more than interferences. In this study, we analyzed the dataset composed of a 4 × 2 cross-over design: exercise mode 4 levels (walking, resistance exercise, badminton, and seated rest as a control condition) × time 2 levels (pre- and post-exercise).

## Materials and Methods

### Participants

The sample size was calculated using power analysis for a one-way repeated ANOVA with partial eta squared (η_*p*_^2^) of 0.05, power (1–β) of 0.95, expected ICC of.50, and α at 0.05. This analysis indicated the sample size was 43 adequate. Participants consisted of undergraduate students from Tohoku Gakuin University who volunteered to participate in the study. A total of 48 healthy participants (25 men, 23 women) were included in the final analysis. All participants were determined to be free of any cardiopulmonary and metabolic disease and visual disorder. The participants were asked to refrain from alcohol use and strenuous physical activity for 24 h before each experiment, and from smoking, food or caffeine consumption for 2 h preceding the experiments. Written informed consent was obtained from all participants before the first experiment. The Human Subjects Committee of Tohoku Gakuin University approved the study protocol. Table 1 shows the characteristics of the participants.

**TABLE 1:** Characteristics of participants (Mean ± SE).

Variables	Total (*N* = 48)	Men (*N* = 25)	Women (*N* = 23)
Age (years)	20.5 ± 0.2	20.7 ± 0.2	20.3 ± 0.2
Height (cm)	165.6 ± 1.4	173.6 ± 0.8	156.8 ± 0.9
Weight (kg)	62.8 ± 2.2	73.5 ± 2.4	51.1 1.4
BMI (kg·m^–2^)	22.7 ± 0.6	24.4 ± 0.9	20.8 ± 0.5
$\dot{\text{V}}$O_2_peak (ml·kg^–1^·min^–1^)	46.5 ± 1.0	50.2 ± 1.2	42.6 ± 1.2
HRpeak (bpm)	196.2 ± 1.1	197.3 ± 1.4	194.9 ± 1.8
10-RM chest press (kg)	32.1 ± 1.9	42.0 ± 2.2	21.4 ± 0.8
10-RM seated row (kg)	37.2 ± 1.8	46.2 ± 2.1	27.5 ± 0.9
10-RM leg press (kg)	70.8 ± 4.0	93.0 ± 3.9	46.7 ± 1.7

### Procedure

#### Day 1

Participants were required to visit the sports physiology laboratory in the gymnasium on five different days (average interval, 4.5 ± 1.6 days). During the first visit, each participant received a brief introduction to this study and completed informed consent. Their height and weight were measured using a stadiometer and a digital scale, respectively. Next, a Stroop/reverse-Stroop color-word test (Hakoda and Sasaki, 1990) was administered to familiarize participants with the test. A fitness assessment that measured 10-repetition maximum (RM) of 3 resistance exercises (chest press, seated row, and leg press) and aerobic fitness (peak oxygen uptake: $\dot{\text{V}}$O_2_peak) was then conducted.

#### Day 2–5 Experimental Sessions

Laboratory visits 2 to 5 were experimental sessions. Participants completed 4 treatment interventions (walking, resistance exercise, badminton, and seated rest). To minimize the learning effect on the Stroop/reverse-Stroop test, the orders of experimental sessions were counterbalanced. We then confirmed there was no bias between order and exercise mode [χ^2^(9) = 2.3, *p* = 0.985]. After arriving at the laboratory, participants rested on a comfortable chair for 10 min, then they wore a heart rate (HR) monitor (Model RS800cx; Polar Electro Oy, Kempele, Finland). Before and after each intervention, participants lay on a bed for 5 min to calm their HR, then completed the Stroop and reverse-Stroop test. HR was monitored throughout experimental session, oxygen uptake ($\dot{\text{V}}$O_2_) was also measured by a portable indirect calorimetry system (MetaMax-3B; Cortex, Leipzig, Germany) during each intervention for 10 min. HR and $\dot{\text{V}}$O_2_ were averaged for last 7 min.

During the walking condition, walked briskly on a motor-driven treadmill (O2road, Takei Sci. Instruments Co., Niigata, Japan). The speed of brisk walking was set at 6.0 km⋅h^–1^. Participants were instructed to walk at a brisk but comfortable pace. However, none changed their speed, and all participants completed the brisk walking at the initial speed. During the resistance exercise, participants performed least two sets of 10 repetitions at 10-RM for three exercises (chest press, seated row, and leg press) using a series of machines (Life Fitness Pro2 series models, Life Fitness, IL) in the gym adjacent to the laboratory. Participants were given a 30 s rest between each set and exercise. During the badminton condition, participants played a singles game against one of three experimenters who had experience in instruction of badminton in the arena adjacent to the laboratory. The investigators played at a level of proficiency that matched the participant’s level and also provided the participants with advice for improvement during the games. During the game, the scores were not recorded and “victory or defeat” was not determined. During the control intervention, participants were seated on a comfortable chair with their smart phones and were instructed to spend time operating their smartphones as normal.

### Physical Fitness Assessment

Participants performed a graded exercise test on the motor-driven treadmill. The initial speed was set 7.2–9.6 km⋅h^–1^ according to estimated physical fitness levels of each participant. Each stage lasted 2-min and was increased by 1.2 km⋅h^–1^ per stage until volitional exhaustion occurred. $\dot{\text{V}}$O_2_ was measured throughout the test (MetaMax-3B) and the average of the final 30 s was defined as the $\dot{\text{V}}$O_2_peak. HR was monitored throughout the test, and rating of perceived exertion (RPE) was taken at the end of each stage.

To determine the load of the resistance exercise, 10-RM for chest press, seated row, and leg press were measured using the weight stack machines. After warm-up trials, following the advice of an instructor, participants performed 10 repetitions at an initial load selected by participant’s perceived capacity for the 3 exercises. After a 3 min rest, participants performed 10 more repetitions at a load adjusted by the participant based on their perception of the previous set. Participants selected the load of the resistance exercise from one of the two sets closest to the 10-RM.

### Stroop and Reverse-Stroop Task

The Stroop/reverse-Stroop test is a pencil and paper exercise that requires manual matching rather than oral naming of items. It consists of four tests arranged in the following order: First is the reverse-Stroop neutral test. Here, a color name (e.g., red) in black ink is in the leftmost column and five different color patches (red, blue, yellow, green, and black) are placed in right side columns. Participants are asked to check the patch corresponding to the color name. Second is the reverse-Stroop incongruent test. Here, a color name (e.g., red) is written in colored ink (e.g., blue) in the leftmost column and five different color patches are in the right-side columns. Participants are instructed to check the patch corresponding to the color name in the leftmost column. Third is the Stroop neutral test. Here, a color patch (e.g., red) is in the leftmost column and five different color names in black ink are in the right-side columns. Participants are asked to check the color name corresponding to the color patch in the leftmost column. Forth is the Stroop incongruent test in which a color name (e.g., red) written using a colored ink (e.g., blue) is in the leftmost column and five color names in black ink are in the-right side columns. Participants are instructed to check a word corresponding to the color of the word in the leftmost column. Each test consists of 100 items and the materials are printed on an A3-size paper. Each test includes practice trails (10 items in 10 s) that precede each test. In each test, participants were instructed to check as many correct items as possible in 60 s. We measured the number of correct responses in each test and then calculated the Stroop- and reverse-Stroop-interferences by subtracting the number of correct responses in the incongruent test from those in the neutral test. Hakoda and Sasaki (1990) recommended the interference ratio (incongruent test score—neutral test score/neutral test score) because the value of the difference between the neutral test score and the incongruent test score for the inhibitory function varies depending on the neutral test score when investigating inhibitory function in a cross-sectional study. However, we employed the interference (incongruent test score—neutral test score) for two reasons. One reason is that both the interference and the interference ratio are substantially equal in a well-controlled longitudinal study that compares the inhibitory function changes over time-course. In practice, we confirmed that there were extremely high correlation coefficients between the interference ratio and the interference divided into each exercise mode and time (pre-, and post-test) (Reverse-Stroop task: *r* ≥ 0.937; Stroop task *r* ≥ 0.978). The other reason is that several previous reliability studies used the interference (Strauss et al., 2005; Hedge et al., 2018; Parsons et al., 2019). Therefore, we feel the interference can provide more relevant information than the interference ratio.

### Statistical Analysis

All measurements were described as group mean ± standard error. Statistical analyses were conducted using IBM SPSS 25 (SPSS Inc., Chicago, IL, United States). To examine the exercise intensity of each intervention, %$\dot{\text{V}}$O_2_peak and %HRmax were compared by the LMM with exercise mode as a fixed effect and participant as a random effect. A significant main effect of exercise mode was followed up with the Bonferroni method.

To calculate the ICC of the performance of each the Stroop, reverse-Stroop test, and the interferences throughout the whole of interventions, the following statistical model in the LMM was used.

$$y_{ijk}=\mu +\alpha_{j}+\beta_{k}+{\left(\alpha \beta \right)}_{jk}+b_{i}+{\left(b\alpha \right)}_{ij}+{\left(b\beta \right)}_{ik}+e_{ijk}$$

where, *y*_*ijk*_ is the number of correct responses in each test or the Stroop or reverse-Stroop interferences of participant *i* = 1,…, *I* observed in the exercise mode *j* = 1,…, *J* at time point *k* = 1,…, *K*, with μ the grand mean, α*_*j*_* the fixed effect of the exercise mode, β*_*k*_* the fixed effect of time, (αβ)*_*jk*_* the fixed effect of the interaction of exercise mode and time, *b*_*i*_ ∼ *N*(0, σ*_*p*_*^2^) the random effect of participant, (*b*α)_*ij*_ ∼ *N*(0, σ_*pm*_^2^) the random effect as the interaction of participant and exercise mode, (*b*β)_*ik*_ ∼ *N*(0, σ_*pt*_^2^) the random effect as the interaction of participant and time, and e*_*ijk*_* ∼ *N*(0, σ*_*e*_*^2^) the residual. The REML was used to estimate parameters. The structure of the random effects was assumed as variance components. Following the manner by Brouwer et al. (2012) and Demetrashvili et al. (2016), the ICC was calculated by following equation.

$$ICC=\frac{\sigma_{p}^{2}+\sigma_{pm}^{2}+\sigma_{pt}^{2}}{\sigma_{p}^{2}+\sigma_{pm}^{2}+\sigma_{pt}^{2}+\sigma_{e}^{2}}$$

In Equation 2, the numerator is a sum of the random effects concerned with individual differences, and the denominator is the sum of the random effects and the residual variance. If individual performance is consistent throughout the whole experiment, the ICC should be high. We then calculated a 95% confidence interval of the ICC using the *F*-approach by Demetrashvili et al. (2016). Based on Shrout (1998), we assessed ICCs as follows: “substantial” is 0.81–1.00; “moderate” is 0.61–0.80; “fair” is 0.40–0.60; “slight” is 0.10–0.40; “virtually none” is 0.0–0.10. To investigate the fixed effects, if the interaction (exercise mode × time) was significant in the LMM model, another LMM model, in which a fixed effect is exercise mode and a random effect is participant, and the Bonferroni methods were conducted for pre-test and post-test, respectively.

## Results

### Intensity of Interventions

Table 2 represents intensities of each intervention. The results of the LMM for %$\dot{\text{V}}$O_2_peak and %HRpeak revealed significant main effects [*F*(3, 141) ≥ 276.2, *p* < 0.001], badminton showed significantly higher %$\dot{\text{V}}$O_2_peak and %HRpeak than other interventions (*p* < 0.001, Cohen’*d* ≥ 1.59). The seated rest showed significantly lower %$\dot{\text{V}}$O_2_peak and %HRpeak than the other interventions (*p* < 0.001, Cohen’*d* ≥ 3.53). Differences of %$\dot{\text{V}}$O_2_peak and %HRpeak between the walking and resistance exercise were not significant (*p* ≥ 0.056, Cohen’*d* ≤ 0.438).

**TABLE 2:** Intensities of each intervention (Mean ± SE).

Variables	Intervention	Mean ± SE
%$\dot{\text{V}}$O_2_peak (%)	Walking	45.2 ± 1.4^a^
	Resistance exercise	41.3 ± 1.0^a^
	Badminton	74.3 ± 1.6^a,b,c^
	Control	9.9 ± 0.3^b,c,d^
%HRpeak (%)	Walking	60.2 ± 1.3^a^
	Resistance exercise	64.3 ± 1.4^a^
	Badminton	79.2 ± 1.3^a,b,c^
	Control	35.4 ± 0.6^b,c,d^

*^*a*^Significant difference from Control, p < 0.05 adjusted by Bonferroni method. ^*b*^Significant difference from Aerobic exercise, p < 0.05 adjusted by Bonferroni method. ^*c*^Significant difference from Resistance exercise, p < 0.05 adjusted by Bonferroni method. ^*d*^Significant difference from Badminton, p < 0.05 adjusted by Bonferroni method.*

### Fixed Effects on Cognitive Performances

Table 3 represents each test performance and interference across exercise mode and time. The LMM showed significant interactions for the reverse-Stroop neutral test [*F*(3, 141) = 3.9, *p* = 0.010] and the Stroop incongruent test [*F*(3, 188) = 5.5, *p* = 0.001]. Results of the *post hoc* analysis indicate that while no main effects of exercise mode were revealed on pre-test for both of the reverse-Stroop neutral and Stroop incongruent test [*F*(3, 141) < 0.3, *p* > 0.814], significant main effects of exercise mode were found on post-test for both the reverse-Stroop neutral test and Stroop incongruent test [*F*(3, 141) > 3.2, *p* ≤ 0.026]. Badminton significantly enhanced performance of the reverse-Stroop neutral test (*p* = 0.018, Cohen’s *d* = 0.378) and the Stroop incongruent test (*p* = 0.006, Cohen’s *d* = 0.369) relative to control. For the reverse-Stroop incongruent and Stroop neutral tests, although there were no significant interactions [*F*(3, 188) < 2.0, *p* ≥ 0.111] and main effects of exercise mode [*F*(3, 141) < 1.7, *p* ≥ 0.161], main effects of time were significant [*F*(3, 188) > 22.3, *p* < 0.001]. For the Stroop and reverse-Stroop interferences, main effects of exercise mode [*F*(3, 141) ≤ 0.9, *p* ≥ 0.425] and time [*F*(1, 47) ≤ 2.0, *p* ≥ 0.162], and interactions [*F*(3, 141) ≤ 2.4, *p* ≥ 0.067] were not significant.

**TABLE 3:** Each test of the Stroop and reverse-Stroop tasks (Mean ± SE) across exercise modes and time.

Task	Exercise mode	Neutral test		Incongruent test		Interference	
		Pre-test	Post-test	Pre-test	Post-test	Pre-test	Post-test
Stroop task	Walking	54.3 ± 1.1	56.5 ± 1.0	50.5 ± 1.2	52.6 ± 1.2	3.7 ± 0.5	3.9 ± 0.7
	Resistance	53.9 ± 1.1	57.3 ± 1.0	50.4 ± 1.3	52.7 ± 1.1	3.4 ± 0.5	4.7 ± 0.7
	Badminton	54.7 ± 1.1	57.7 ± 0.9	50.3 ± 1.2	54.8 ± 1.1*	4.4 ± 0.7	2.9 ± 0.7
	Control	54.2 ± 1.0	55.7 ± 1.1	50.1 ± 1.2	51.8 ± 1.2	4.2 ± 0.7	3.9 ± 0.6
Reverse-Stroop task	Walking	74.9 ± 1.2	77.6 ± 1.2	61.6 ± 1.2	62.6 ± 1.2	13.4 ± 0.7	15.0 ± 1.0
	Resistance	74.5 ± 1.3	78.2 ± 1.2	61.5 ± 1.2	64.4 ± 1.4	13.0 ± 0.9	13.8 ± 1.0
	Badminton	75.5 ± 1.2	79.5 ± 1.1*	62.5 ± 1.0	65.2 ± 1.2	13.0 ± 0.7	14.4 ± 1.0
	Control	75.5 ± 1.4	76.5 ± 1.2	60.5 ± 1.3	62.2 ± 1.3	15.0 ± 0.9	14.3 ± 0.8

*Significant difference from Control in pre- and post-test each, p < 0.05 adjusted by Bonferroni method.

### Random Effects on Cognitive Performances

When the LMM were conducted for the Stroop and reverse-Stroop tasks, it appeared that the variance of the random interaction of the participant and time gradually transited to the random effect of the participant. Finally, the variance of the random interaction of the participant and time calculated as 0.0, indicating that the covariance parameter was redundant. Yamazaki et al. (2018) reported that individuals with a lower performance before exercise tend to increase greatly in performance after exercise. The results of Yamazaki et al. (2018) implies that there might be a multiple co-linearity between the random effect of the participant and the random interaction of the participant and time. The multiple co-linearity might cause redundant random interactions. Therefore, we modified the model by removing the redundant parameter from the models.

**FIGURE 1:**
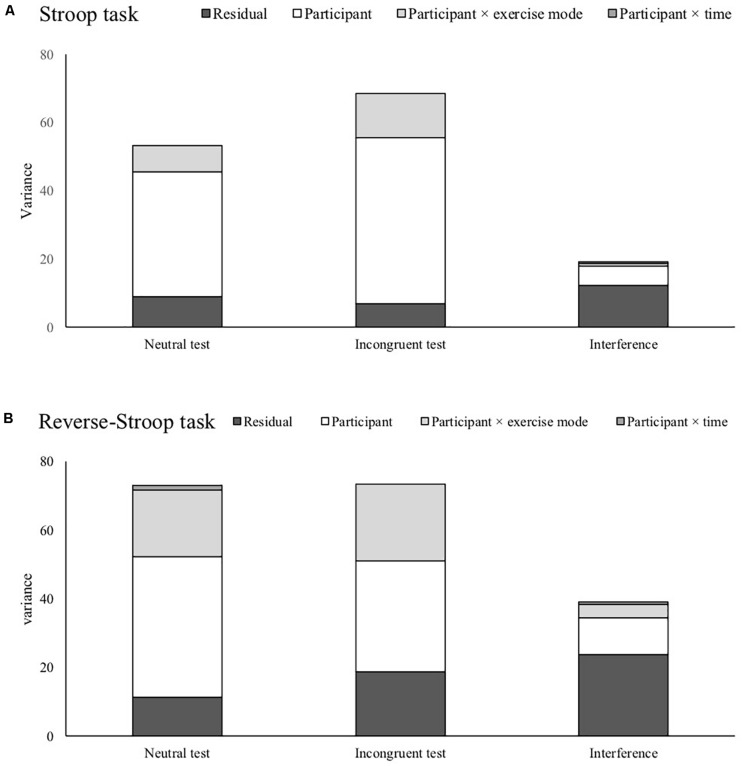
Comparison of variances concerned with participants individuality and the residual variance for the Stroop task **(A)** and the reverse-Stroop task **(B)**.

Figure 1 shows each random effect and the residual across each test condition. For the Stoop and reverse-Stroop task, while there were no differences in the residual in all of the indices, random effects in the interferences became much smaller than the neutral and incongruent test. Table 4 shows the ICC for each test and interference. The ICCs of all tests were more than “moderate” ICCs (ICC ≥ 0.745). Notably, reverse-Stroop neutral test, Stroop neutral test, and Stroop incongruent test showed “substantial” ICC (ICC ≥ 0.833). On the other hand, the ICCs of both the reverse-Stroop interference (ICC = 0.392) and the Stroop interference (ICC = 0.362) were “slight” ICC.

**TABLE 4:** The intraclass correlation coefficients (ICCs) and 95% confidence intervals (95% CI) for each test and interference.

Test/Interference	ICC (95% CI)
Stroop neutral test	0.833 (0.761–0.882)
Stroop incongruent test	0.901 (0.856–0.931)
Stroop interference	0.362 (0.213–0.504)
Reverse-Stroop neutral test	0.846 (0.782–0.890)
Reverse-Stroop incongruent test	0.745 (0.661–0.810)
Reverse-Stroop interference	0.392 (0.247–0.527)

## Discussion

This study investigated ICCs of the Stroop and reverse-Stroop tasks in a cross-over research design. The main finding of this study was that different results were found in the Stroop tests and interference. There was the significant interaction of exercise mode and time for the Stroop incongruent test, while the LMM did not reveal a significant interaction for the Stroop neutral test. The *post hoc* analysis for the incongruent test revealed that the badminton selectively enhanced the incongruent test performance compared with the control, suggesting that the effects of acute exercise on inhibitory function are influenced by exercise modes. The results that the badminton, which is a hard intensity and open-skilled exercise, improves cognitive functions more than a light intensity and closed-skilled exercise agree with the results of systematic reviews (Chang et al., 2012; Gu et al., 2019). There were also large random effects associated with participants comparing with the residual variance for the Stroop tests. The large random effects and small residual yielded “substantial” ICCs throughout the whole experimental procedure, suggesting that the Stroop tests are highly reliable measures for cross-over design researches.

In contrast to the Stroop tests, the LMM did not reveal fixed effects concerned with exercise modes on inhibitory function for the Stroop interference. The Stroop interference also showed much lower ICC relative to both the Stroop tests. These results suggest that calculation of the interference might attenuate the individual differences as the numerator of ICC, resulting in low reliability and statistical power. Given these results, for cross-over design investigating how acute exercise benefits inhibitory function, analyzing the performances of the Stroop neutral/congruent and incongruent tests separately and comparing their changes might be a better approach than calculating and analyzing the Stroop interference. The Stroop interference is known as a valid measure for inhibitory function for cross-sectional studies (Takeuchi et al., 2012; Byun et al., 2014; Song and Hakoda, 2015; Fagundo et al., 2016; Scarpina and Tagini, 2017). However, because of the possibility of low reliability and statistical power with the Stroop interference, employing Stroop interference as a dependent variable could reduce the likelihood of finding the effects of exercises for cross-over design study.

The reverse-Stroop test showed different results from the Stroop tests about the fixed effects. While the LMM found a significant interaction of exercise mode and time for the neutral test, there was no significant interaction for the incongruent test. We also did not find significant effects of exercise mode, time and interaction for reverse-Stroop interference. These results suggest that there is no effect of acute exercise on inhibitory function measured by the reverse-Stroop task. We expected that the reverse-Stroop task would be more sensitive to an effect of acute exercise because the previous studies (Tsukamoto et al., 2016a,b) showed that the reverse-Stroop incongruent test and the reverse-Stroop interference were significantly enhanced by acute exercises. There is a possibility that the different measurement methods between the previous studies and the present study seems to cause different results. The previous studies (Tsukamoto et al., 2016a,b), employing small sample sizes (*N* = 12 and *N* = 10, respectively), measured the Reverse-Stroop neutral and incongruent tests by a computerized test. They found large significant effects of acute exercise on the Reverse-Stroop interference ratio. Although the effect sizes for the previous studies (e.g., Cohen’s *d* or partial η square) were not reported, considering the small sample size, we expected that the Reverse-Stroop tests would be more sensitive to the effect of acute exercise. However, in spite of the relatively large sample size (*N* = 48), unexpectedly, the LMM did not reveal any effects of exercise on the Reverse-Stroop tests measured by a pencil and paper method in the present study. Given that the effect of exercise on the Stroop tests in the present study is similar to the systematic reviews (Chang et al., 2012; Gu et al., 2019), the difference between computerized test and pencil and paper test might be a critical factor in the Reverse-Stroop task.

Although the LMM showed differences in fixed effects among the Stroop and reverse-Stroop tests, Random effects and ICCs for the reverse-Stroop tests were similar to the Stroop tests. The neutral test and incongruent test for the reverse-Stroop task showed larger random effects concerned with individual differences relative to the residuals, resulting in more than “moderate” ICCs. The results suggest that the two reverse-Stroop tests are reliable measurements as well as the Stroop tests. The changes of random effects for the reverse-Stroop task from each test to the interference were also similar to the Stroop task. For the reverse-Stroop interference, random effects concerned with individual differences vastly decreased compared with those of the neutral and incongruent tests. Still, the residuals did not much differ from each test to the interference. This discrepancy of changes for random effects and residual seems to be the leading cause of the low reliability of the interferences for the cross-over design.

The comparison of each variance across tests and interferences revealed that the main reason for reduced ICC for the interferences was due to the reduction of random effects concerned with individual differences. These results strongly support our hypothesis that the Stroop and reverse-Stroop tests show higher ICCs than the interferences. Given the small to moderate effect of exercise on cognitive function (Lambourne and Tomporowski, 2010; Voss et al., 2011), experimental studies investigating how exercise benefits inhibitory function, employing the interferences for the Stroop and reverse-Stroop tasks with low reliability as a dependent variable might mask the significance of the effect of an acute exercise. The Stroop and the reverse-Stroop incongruent test appear to be affected by inhibitory function and information processing. Therefore, interference that partial out the influence of information processing by subtracting the neutral/congruent tests from the incongruent test might be a reasonable method of assessment. Indeed, substantial cross-sectional studies employed interference to investigate the association between interferences and brain structure or behavioral measurements (Takeuchi et al., 2012; Fagundo et al., 2016; Peven et al., 2018). However, several experimental studies which detected a selective effect of interventions on inhibitory function have used the incongruent test as the dependent variable (Ferris et al., 2007; Nouchi et al., 2013; Ishihara et al., 2017). The results of this study might explain why the previous experimental studies used the Stroop or reverse-Stroop incongruent test not but interference. It seems that interference with “slight” ICC is not sensitive to the impact of exercise or any factors (i.e., time ore learning effect). Given more than “moderate” ICCs of the neutral and incongruent tests for the Stroop and reverse-Stroop tasks, analyzing the neutral and the incongruent tests, respectively, and comparing outputs of the analyses for both of the Stroop tasks also might be a better approach to understand comprehensively how acute exercise works on inhibitory function.

## Limitation

One notable difference between the present study and previous research is in the measurement method. We used a paper and pencil matching test to measure each performance of the Stroop and reverse-Stroop task, showing that the calculation of interference for the Stroop and reverse-Stroop tasks decreases the ICC and might mask the fixed effects in cross-over design research. These results and our interpretation correspond to most of the previous studies that measured the Stroop and reverse-Stroop tasks in their experiments. Other studies were detected the fixed effects by analyzing the Stroop interference (Hyodo et al., 2012; Byun et al., 2014) and reverse-Stroop interference (Tsukamoto et al., 2016a,b). Particularly, the difference in measurement methods might selectively influence the performance of Reverse-Stroop tasks. As described above, we had expected that Reverse-Stroop tasks would be sensitive to exercise based on previous studies (Tsukamoto et al., 2016a,b) that showed the Reverse-Stroop performance measured by a computerized test is extremely sensitive to exercise. However, we did not find any effects of exercise on the Reverse-Stroop tests in the present study. This inconsistency between the present study and previous studies might be due to differences between a computerized test and a pencil and paper test. There are fewer studies that have used Reverse-Stroop tasks relative to Stroop tasks, so that we could not interpret that inconsistency about Reverse-Stroop tasks. Therefore, other measurement methods, such as a computerized test or an oral test, might change the influence of calculation of the interference on the ICC. To clarify an interaction between test manners and types of cognitive function, further studies would be needed in the future.

## Conclusion

In conclusion, the performance of each neutral and incongruent test for the Stroop and reverse-Stroop tasks has a high ICC while calculating the interference decreases ICC in cross-over design research. We have shown that the cause of the decrease of ICC is the reduction of variances associated with individual differences. The interference for the Stroop and reverse-Stroop tasks are valid indices for the inhibitory function. However, to investigate the effect of exercise on the inhibitory function with adequate statistical power in cross-over design research, researchers should also draw attention to incongruent test performance for the Stroop and reverse-Stroop tasks.

## Data Availability Statement

The original contributions presented in the study are included in the article/Supplementary Material, further inquiries can be directed to the corresponding author/s.

## Ethics Statement

The studies involving human participants were reviewed and approved by the Human Subjects Committee of Tohoku Gakuin University. The patients/participants provided their written informed consent to participate in this study.

## Author Contributions

ST: conception of this research, data collection, analysis and interpretation, and writing original draft. PG: supervision and review and editing. Both authors contributed to the article and approved the submitted version.

## Conflict of Interest

The authors declare that the research was conducted in the absence of any commercial or financial relationships that could be construed as a potential conflict of interest.
